# Optimization of a Plasma Rich in Growth Factors Membrane for the Treatment of Inflammatory Ocular Diseases

**DOI:** 10.3390/bioengineering9100508

**Published:** 2022-09-27

**Authors:** Eduardo Anitua, María de la Fuente, Jesús Merayo-Lloves, Francisco Muruzabal

**Affiliations:** 1 Regenerative Medicine Laboratory, Biotechnology Institute (BTI), 01007 Vitoria, Spain; 2 Instituto Oftalmológico Fernández-Vega, Fundación de Investigación Oftalmológica, Universidad de Oviedo, 33012 Oviedo, Spain

**Keywords:** plasma rich in growth factors, platelet rich plasma, PRP, ocular surface diseases, autoimmune diseases, complement system, heat inactivation, fibrin membrane

## Abstract

The main purpose of the present study is to develop an immunosafe fibrin membrane obtained by plasma rich in growth factors technology (is-mPRGF) with improved mechanical properties that could be applied in patients with inflammatory ocular diseases. Blood was drawn from three healthy donors and centrifuged, and the collected PRGF was activated and distributed into two groups: (i) mPRGF: a PRGF membrane maintained at 37 °C for 30 min; (ii) IS5+30: mPRGF incubated at 37 °C for 5 min and then incubated at 56 °C for 30 min. The content of both membranes was analyzed for several growth factors such as IgE and the complement activation, as well as biological activity on different ocular surface cells. Furthermore, the physical and mechanical characterizations were also evaluated. IS5+30 completely reduced the complement activity and decreased the IgE while preserving the concentration of the main growth factors. IS5+30 induced similar biological activity regarding mPRGF on the different ocular surface cells analyzed. Furthermore, no significant differences in release kinetics or fibrin degradation were observed between both membranes. Summarizing, IS5+30 totally reduces complement activity while preserving the concentration of most growth factors and their biological activity. Furthermore, the physical and mechanical properties of the fibrin membrane are preserved after heat inactivation.

## 1. Introduction

Over the last 3 decades, platelets were ascertained as one of the main actors in the tissue regenerative scene as the high content of growth factors mainly present in their alpha granules was revealed [1,2]. This encouraged the idea that an increase in platelet concentration would induce an improvement in the regeneration of damaged tissues. This idea paved the way for the development of several protocols and procedures to obtain a type of blood-derived product designated platelet-rich plasma (PRP) because of its enrichment in platelet concentration with respect to the concentration of platelets in peripheral blood [3]. Since then, different types of PRP have emerged with different characteristics in terms of platelet concentration, presence or absence of leukocytes and erythrocytes, and platelet activation or not [4,5].

Plasma rich in growth factors (PRGF) is a type of PRP with specific characteristics that make it different from other PRPs. Some of these attributes are related to its platelet concentration which ranges between 1.5 and 2.5 times the platelet concentration over peripheral blood, the absence of leukocytes and erythrocytes, and its activation with calcium chloride. Furthermore, one of the main characteristics of PRGF technology is its versatility since several formulations can be obtained from the same initial product [6]. After activation with calcium chloride, an injectable formulation can be obtained which has been used for the treatment of various pathologies in different medical fields such as dentistry, dermatology, and traumatology [7,8]. Moreover, if PRGF is incubated for 40 to 60 min at 37 °C after platelet activation, a supernatant rich in growth factors is obtained [9]. The latter formulation has been used as eye drops in the ophthalmology field for the treatment of several ocular diseases such as dry eye, persistent epithelial defects, or corneal ulcers [10,11,12]. Finally, one of the most interesting formulations that can be obtained with PRGF technology is a fibrin membrane and fibrin clot [13]. Both have been used for the treatment of deep injured tissues or to incorporate a scaffold for facilitating the healing of large, damaged tissue areas [14,15].

On the other hand, a personalized PRGF eye drop formulation, called immunosafe PRGF eye drops, has been recently developed with several immunological components diminished such as complement activity and IgE [16]. These eye drops were obtained after activation with calcium chloride and incubation at 37 °C for at least 40 min and then incubated at 56 °C for 30 to 60 min. The results obtained in that study revealed that the main proteins and growth factors involved in tissue regeneration as well as the biological activity of PRGF were preserved after PRGF heat inactivation. This customized product was developed for use in the treatment of several eye diseases whose etiopathogenesis involves various inflammatory processes, such as dry eye disease in patients with systemic immune disorders or autoimmune diseases such as Sjögren’s syndrome or graft-versus-host disease [17,18]. However, in some clinical practices, it is necessary to apply a scaffold-like PRGF fibrin membrane to improve the ocular tissue regeneration [19]. In those cases where the patient suffers from an autoimmune disease or where there is an inflammatory component involved in its etiopathology, it would be necessary to use a scaffold with reduced immunogenicity to treat these types of patients.

In the present study, we propose the development of an immunosafe PRGF membrane (is-mPRGF) with improved mechanical properties that could be applied to patients with ocular surface diseases whose etiopathogenesis is associated with an inflammatory component. This is-mPRGF membrane was obtained after a combined heat treatment at 37 °C and 56 °C. We hypothesized that heat treatment of mPRGF at 56 °C would inactivate the complement system while maintaining its protein content and biological characteristics. Based on this premise, the levels of various growth factors, proteins, and the complement activity were analyzed. Furthermore, the proliferative potential of this new heat-treated PRGF scaffold was evaluated for different cells on the ocular surface.

## 2. Materials and Methods

### 2.1. Obtaining of PRGF Membranes

Three healthy donors were recruited to provide the blood needed to obtain the different PRGF membranes used in the study. Briefly, after informed consent, blood was collected into 9-mL tubes containing 3.8% sodium citrate as an anticoagulant. The principles of the Declaration of Helsinki were followed to carry out the present study. After centrifugation, the whole column of plasma was collected whilst avoiding the buffy coat using an Endoret ophthalmology kit (BTI Biotechnology Institute, S.L., Vitoria, Spain). Then, the total PRGF volume collected from each donor was aliquoted. For the present study, several aliquots from each donor were collected and distributed into two groups: (i) half of the aliquots were activated with calcium chloride and incubated for 30 min at 37 °C (mPRGF), and (ii) the other half were activated with calcium chloride and incubated for 5 min at 37 °C and for 30 min at 56 °C. This heat-inactivated mPRGF was termed IS5+30.

The dataset of the mPRGF samples used for the comparison with PRGF IS5+30 was employed to obtain the results of a study published previously, in which the mPRGF dataset was compared with the dataset obtained using an immunosafe PRGF membrane obtained after activation with calcium chloride and immediately incubated at 56 °C for 30 min [20].

### 2.2. Evaluation of Proteins and Growth Factor

#### 2.2.1. Analysis Concentration of Different Growth Factors

The concentration of several growth factors, such as platelet-derived growth factor-AB (PDGF-AB), epidermal growth factor (EGF), vascular endothelial growth factor (VEGF), transforming growth factor β1-(TGF-β1), endostatin (END), angiopoietin-1 (ANG-1), and thrombospondin-1 (TSP-1) were measured to analyze the mPRGF after heat treatment. The different growth factors were quantified using ELISA kits from R&D Systems (Minneapolis, MN, USA). To assess the increase or decrease in protein concentration between both PRGF membranes developed in the present study, the final data were expressed as the percentage of the concentration of a certain protein with respect to the mean value obtained for that protein in the mPRGF group.

#### 2.2.2. IgE and Complement Activity Measurement

The evaluation of the total classical complement activity was analyzed by the CH50 assay kit (Wako Chemicals, Richmond, VA, USA). A CH50 assay was performed following the manufacturer’s instructions. On the other hand, to analyze the concentration of immunoglobulin E (IgE) in the PRGF membranes (mPRGF and IS5+30), a particle-enhanced turbidimetric immunoassay (PETIA) from Biokit (Quantex IgE kit, Barcelona, Spain) was used following the user’s manual. To analyze the increase or decrease in the complement activity and IgE concentration after heat treatment of the PRGF membranes, the final data were expressed as a percentage of the complement activity or IgE concentration in each sample with respect to the mean value obtained in the mPRGF group.

#### 2.2.3. Kinetics of Growth Factor Release

To evaluate the protein release kinetics from mPRGF formulations, several growth factors were measured by ELISA assays. Briefly, mPRGF and IS5+30 were obtained from each donor by adding 2 mL of PRGF into 6-well plates, and the steps described above were followed to obtain each membrane. Subsequently, DMEM/F12 media obtained from Gibco-Invitrogen (Grand Island, NY, USA) was added to each well, and then, the samples were incubated at 37 °C in a CO_2_ incubator for 1 h, and 1, 3, and 7 days. A single membrane from each of the PRGF membranes (mPRGF and IS5+30) and obtained from the three donors was used for the single supernatant collection at each time point. At each study time, supernatant from each well was collected and centrifuged at 500 g for 10 min, and the resulting supernatant was aliquoted and stored at −80 °C until use. Diverse growth factors such as PDGF-AB, EGF, TGF-β1, VEGF, basic fibroblast growth factor (FGFb), ANG-1, and END (R&D System) were analyzed following the protocol handbook. The release kinetics were evaluated throughout the study period with respect to the complete release of each protein or growth factor from the mPRGF obtained from each donor. The percentage of each protein released at each time point was then calculated with respect to the mean concentration of the total protein released from the mPRGF.

### 2.3. Evaluation of Membrane Degradation

To analyze the degradation of the different PRGF membranes obtained in this study, mPRGF and IS5+30 membranes obtained from the different donors were exposed to a tissue plasminogen activator (tPA) for 8 days. tPA is an enzyme which catalyzes the conversion of plasminogen to plasmin, the latter being the main enzyme involved in fibrin degradation. Both PRGF membranes (mPRGF and IS5+30) obtained from each donor and prepared as described above were immersed into 1 mL of PRGF for 24 h at 37 °C to allow for a complete fibrin contraction. Subsequently, each membrane was weighed to obtain the initial weight (W0), and then tPA was added to the milliliter of the PRGF at a concentration of 0.25 μg/mL (AbCam, Cambridge, UK) and then incubation of the samples at 37 °C. The weight of each membrane was evaluated at different time points (1, 2, 3, 6, 7, and 8 days), and the mass remaining percentage of both PRGF membranes was calculated as follows:

mass remaining % = (Wd/W0) × 100

in which W0 was the basal weight of each PRGF membrane, and Wd is the weight of each PRGF membrane at each study time.

### 2.4. In Vitro Cell Studies

#### 2.4.1. Culture of Ocular Surface Cells

The biological activity of each mPRGF and IS5+30 membrane was evaluated using three types of ocular surface cells: (i) human keratocytes (HK) and (ii) conjunctival fibroblasts (HConF) both obtained from ScienCell Research Laboratories (San Diego, CA, USA) and (iii) corneal epithelial cells (HCE) obtained from Riken Cell Bank (RCB1384: HCE-T, Ibaraki, Japan). HCE, HK, and HConF cells were grown using the media and conditions described in the manufacturer’s instructions, similarly as described in previous studies [16].

#### 2.4.2. Proliferative Potential of PRGF Membranes

The proliferative potential of each PRGF membrane was evaluated on HCE, HK, and HConF cells. The mPRGF and IS5+30 membranes were obtained from each donor as described above and squeezed using a membrane former (BTI Biotechnology Institute, Vitoria, Spain). The released supernatants were filtered, aliquoted, and stored at −80 °C until use. HCE, HK, and HConF cells were treated with the different supernatant obtained from each PRGF membrane diluted to 20% (*v*/*v*) in serum-free medium for 72 h. The evaluation of cell proliferation was carried out using the Cyquant cell proliferation assay (Molecular Probes-Life technologies) according to the manufacturer’s protocol. To evaluate the higher or lower proliferative potential of mPRGF and IS5+30 membranes, the final data were expressed as the percentage of proliferation in each well with respect to the mean proliferation value obtained in the mPRGF group. Furthermore, a proliferative assay was performed on different ocular surface cells such as human corneal epithelial cells (HCE), human conjunctival cells (HConF), and keratocyte cells (HK) to assess the biological potential of each supernatant obtained from each membrane and donor at each time point. The proliferative potential of the supernatant released from each mPRGF at each time point was compared with the complete supernatant released from each type of membrane (control).

### 2.5. Statistical Analysis

Data obtained from the different assays were expressed as mean ± SD. To analyze the statistical differences in the results obtained with the different PRGF membranes using the various assays performed in the present work, the paired *t*-test or the Wilcoxon test was used. *p* < 0.05 was considered as the level of statistical significance. All statistical analysis were performed using SPSS v15.0 (SPSS Inc., Chicago, IL, USA).

## 3. Results

### 3.1. Analysis of Proteins and Growth Factors in PRGF Membranes

#### 3.1.1. Complement Activity and IgE Evaluation

**Figure 1:**
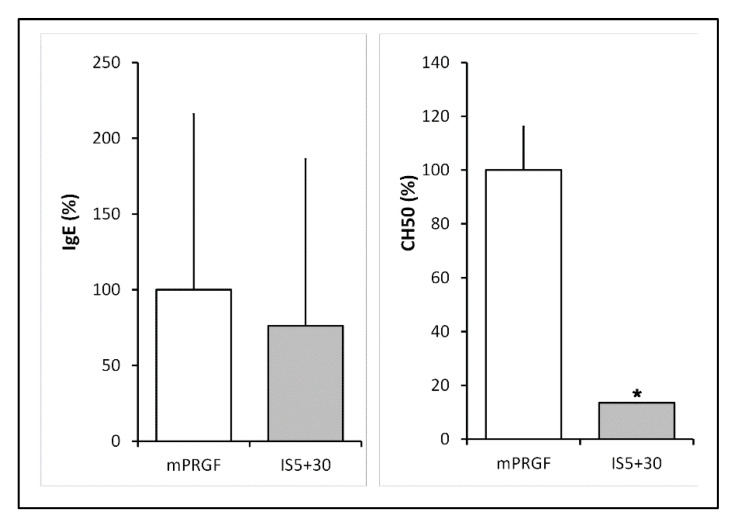
IgE and complement activity levels in non-heat treated (mPRGF) and heat-treated (IS5+30) membranes. IgE levels were reduced by 20% in the heat-treated membrane (IS5+30) regarding mPRGF. No significant differences were observed in the IgE levels between the mPRGF and IS5+30. Heat treatment completely reduces complement activity in the mPRGF incubated at 37 °C for 5 min and at 56 °C for 30 min (IS5+30) compared to the mPRGF. * *p* < 0.05.

Our group previously described the reduction of immunoglobulin E and complement activity during the development of an immunosafe eye drop formulation [16]. In this sense, the IgE and the complement activity levels were quantified to confirm the mPRGF heat inactivation. After heat inactivation of the mPRGF (IS5+30), the IgE levels were reduced by 20% with respect to the mPRGF (Figure 1); however, these differences did not reach statistical significance (*p* > 0.05).

Meanwhile, complement activity (CH50) showed a significant statistical reduction (*p* < 0.05) after heat treatment of the PRGF membrane (IS5+30) compared to the mPRGF (Figure 1). Furthermore, a total inactivation of the complement system in the heat-inactivated mPRGF could be considered because the complement activity values were lower than the detection limits of the assay.

#### 3.1.2. Growth Factor Concentration

**Figure 2:**
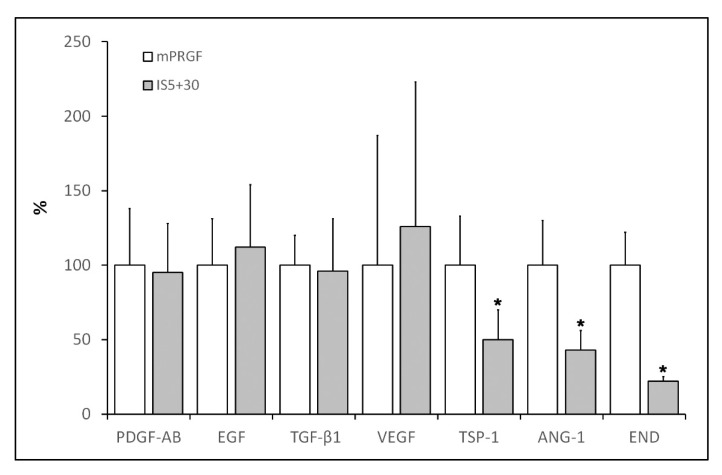
Percentage content of the different growth factors in the IS5+30 membrane with respect to the mPRGF evaluated in the study. Significant reduction (* *p* < 0.05) of TSP-1, ANG-1, and END levels was observed after heat treatment of the PRGF membrane (IS5+30) regarding the non-heated membrane (mPRGF).

The levels of different growth factors and proteins such as EGF, PDGF-AB, TGF-β1, VEGF, TSP-1, ANG-1, and END were analyzed in the supernatant derived from the different membranes obtained in the present study (mPRGF and IS5+30). As shown in Figure 2, mPRGF and IS5+30 showed similar levels of those growth factors that are mainly involved in ocular tissue regeneration such as EGF, PDGF-AB, TGF-β1, and VEGF, while significant differences (*p* < 0.05) in the concentration of angiogenesis-related growth factors such as END, ANG-1, and TSP-1 were found between both PRGF membranes, showing a significant reduction in the concentration of these growth factors in the IS5+30 membranes compared to the mPRGF.

#### 3.1.3. Kinetics of Growth Factor Release

**Figure 3:**
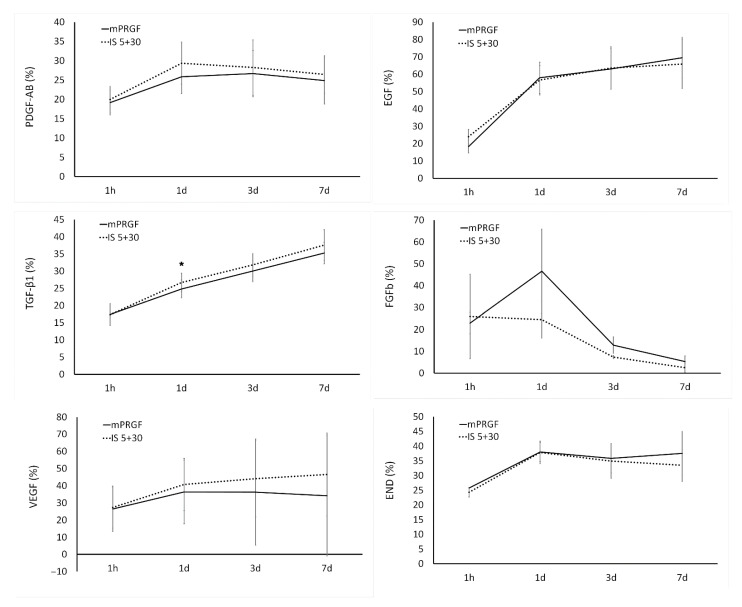
The release kinetics of PDGF-AB, EGF, TGF-β1, FGFb, VEGF, and END were analyzed in both PRGF membranes (mPRGF and IS5+30) for 1 h and 1, 3, and 7 days. Except for TGF-β1 levels at 1d, no significant differences were found between the IS5+30 and mPRGF at any study time points in any of the growth factors evaluated. * *p* < 0.05 significant differences between mPRGF and IS5+30.

The outcomes observed in the release kinetics of the different growth factors analyzed in the present study show that, with the exception of EGF which reached levels close to 70% on day 7, the maximum levels of proteins released from the mPRGF or IS5+30 membranes reached 30% of the total proteins contained in these membranes. The maximum release of most of the proteins analyzed in both types of PRGF membranes was reached on day 1 (Figure 3). For EGF, VEGF, and END, protein release levels were maintained from day 1 to the end of the study period (3 and 7 days). However, the highest release of FGFb was detected on day 1 in the mPRGF, showing after this time point a continuous reduction in its release; however, in the case of the IS5+30 membrane, a steady reduction in the release of this growth factor from the beginning to the end of the study was observed. On the other hand, TGF-β1 release from both membranes (mPRGF and IS5+30) was continuous over the different time points.

For the different growth factors analyzed, the present study only showed statistically significant differences (*p* < 0.05) in TGF-β1 release kinetics on day 1 of the study between both PRGF membranes.

### 3.2. Fibrin Degradation of PRGF Membranes

The mPRGF and IS5+30 membranes were incubated with tPA to analyze the ability of both membranes to be degraded by enzymes that can be found in vivo. The percentage weight loss of the mPRGF and IS5+30 membranes decreased continuously during the different time points analyzed in the study until complete fibrin degradation was reached after incubation with tPA for 8 days. No significant differences were observed between both membranes (mPRGF and IS5+30) throughout the study time (Table 1).

### 3.3. Biological Activity of Both PRGF Membranes

**Figure 4:**
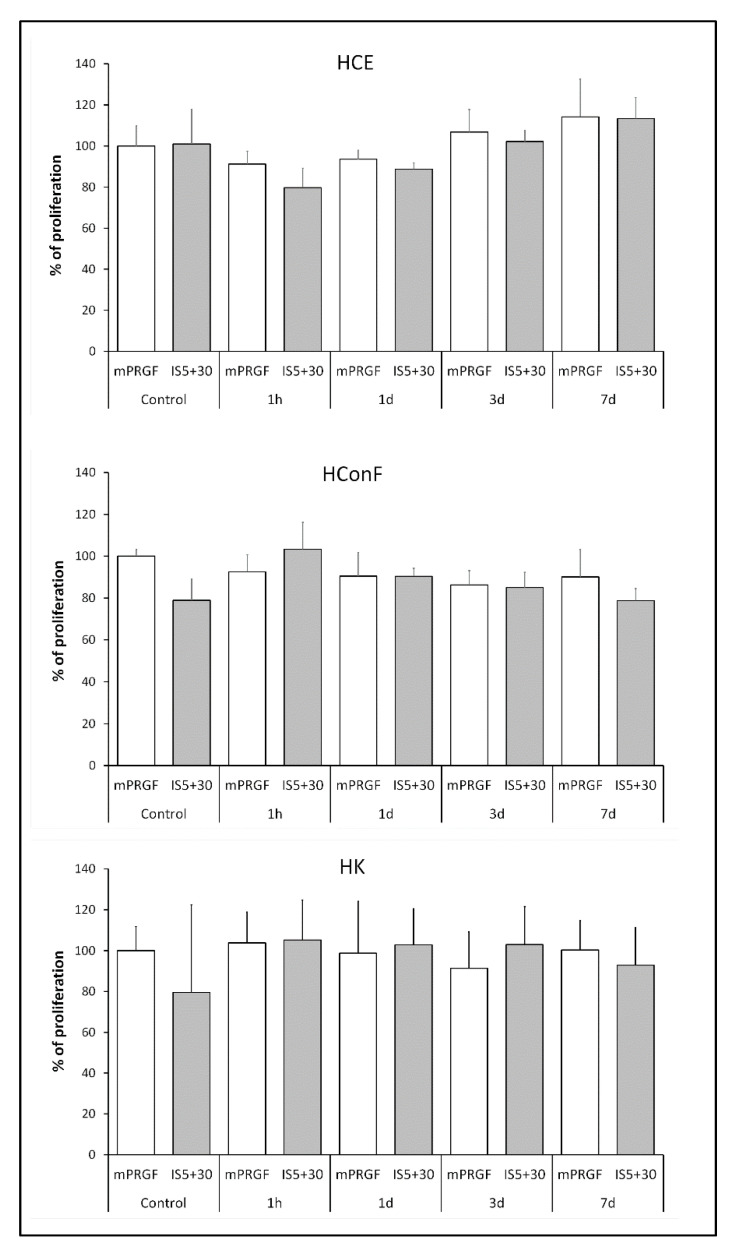
Proliferation percentage of different ocular surface cells (HCE, HConF, and HK) after treatment with the supernatant obtained from each of the PRGF membranes (mPRGF and IS5+30) at each time point of the kinetic release assay (1 h, and 1, 3, and 7 days) compared to the supernatant obtained after complete membrane squeezing of both PRGF membranes (control of mPRGF and IS5+30). No significant differences were observed among the different samples at any study time and in any of the cell types analyzed.

To analyze the biological potential of both membranes developed in the present study, the proliferation of HCE, HK, and HConF cells was evaluated after their incubation with the supernatant obtained from the mPRGF or IS5+30. Both PRGF membranes induced similar proliferative activity in the ocular surface cells. No significant differences were observed between the membranes obtained from the different donors in their induction of proliferation in ocular surface cells (mPRGF and IS5+30 referred as control in Figure 4).

A proliferation assay was performed with the supernatant obtained during the kinetic release study of the mPRGF and IS5+30 membranes acquired from the different donors (Figure 4). The results showed that no significant differences were observed in the proliferative potential of the different supernatants released from both membranes at any of the times studied and the control supernatant obtained from each membrane (whole supernatant released after complete squeezing each membrane type) in any of the ocular surface cells analyzed in the present study (HCE, HConF, and HK).

## 4. Discussion

Ocular tissues have usually been considered to be beyond the reach of the immune system, being in an immuno-privileged situation. However, several studies have demonstrated that this eye condition is frequently disrupted in diverse ocular pathologies in which several immunological components are implicated in the etiopathology of these ocular diseases [21]. In this sense, the immune system has been involved directly or indirectly in a wide range of ocular pathologies of the anterior and posterior chambers of the eye [22]. Dry eye syndromes, autoimmune uveitis, age-related macular degeneration (AMD), glaucoma, and autoimmune retinopathy are some of the anterior and posterior segment diseases in which the immune system plays a main role in their pathological development [21]. Some autoimmune diseases such as rheumatoid arthritis, systemic lupus erythematosus, Sjögren’s syndrome, and graft-versus-host disease are involved in the development of several ocular pathologies mentioned above [22,23]. Although CD4+ T cells have been described in the last two decades as the main actors in the pathogenesis of ocular autoimmune diseases, several studies have demonstrated that B cells and the autoantibodies produced by them also have an outstanding role in the immunopathogenic process of these diseases [23,24]. Furthermore, it has been widely suggested that the complement system is involved in the pathogenesis of several inflammatory ocular diseases through the binding to the autoantibody Fc receptors [25,26,27,28].

In the present study, we describe the development of a fibrin membrane with complete inactivation of the complement system and reduction of IgE concentration after being subjected to a heating process at 56 °C for 30 min and being previously incubated at 37 °C for 5 min to induce fibrin clot formation (IS5+30). It is interesting to highlight that the greatest accomplishment of the present study is the development of a fibrin membrane with improved mechanical characteristics and reduced immunological properties without the loss of its biological activity. The total complement inactivation after heat treatment confers to the new mPRGF (IS5+30) the potential to be used for the treatment of diverse ocular surface disease in patients suffering from autoimmune diseases. In addition, a decrease of around 20% in the IgE content was detected after heat inactivation of the mPRGF without reaching significant differences compared to the non-inactivated mPRGF. However, in the case of patients with ocular disorders resulting from allergic diseases [29], a greater reduction in IgE content could be achieved by increasing the heating time at 56 °C up to 60 min without reducing the content of the main growth factors and proteins involved in ocular tissue regeneration [16].

Furthermore, the results obtained in the present study showed that the new heat inactivated PRGF membrane obtained after incubation at 37 °C for 5 min and at 56 °C for 30 min maintains the concentration of the main proteins and growth factors involved in tissue regeneration compared to the non-inactivated mPRGF. These results were mirrored in the biological activity of the heat-treated mPRGF obtained in this study, where no significant differences in IS5+30 membrane proliferative activity were found in any of the ocular surface cells evaluated in the present study when compared to the non-inactivated mPRGF. Conversely, the levels of some growth factors involved in the angiogenesis process such as angiopoietin-1 (ANG-1) related to angiogenesis activation, and thrombospondin-1 (TSP-1) and endostatin (END) related to the antiangiogenic effect, were significantly diminished after heat inactivation of the mPRGF. It has been overwhelmingly described that upon platelet activation a myriad of proteins and growth factors are released. A wide variety of these biological mediators with similar or counteracting actions are involved in the management of the different stages of regenerative tissue processes such as angiogenesis [30]. Consequently, further studies related to the angiogenic process will be necessary to evaluate the significance of the reduction of this protein after mPRGF heat-inactivation.

Several studies have described a partial denaturalization of plasma proteins, including fibrinogen, after heating inactivation at 56 °C for 1 h [31]. Furthermore, it has been widely described that fibrin clot density and stiffness are directly correlated with fibrinogen concentration but inversely correlated with a clot degradation rate [32,33]. In addition, fibrin clot features such as clot stiffness, microstructure, and resistance to fibrinolysis are defined by the concentration of fibrinogen, thrombin, and FXIIIa which also affect the release kinetics of the different proteins enclosed in the fibrin membrane [34,35]. Likewise, several studies have demonstrated that a fibrin clot with an intermediate or soft stiffness improves the progress of different cell types [36,37,38]. In this case, it is important to highlight that a lower stiffness of the fibrin clot could help tissue regeneration; however, this type of clot may degrade rapidly and may not be beneficial for continuous release of proteins over long periods of time [39].

The fibrin degradation assay carried out in the present study showed that the heat inactivated PRGF membrane (IS5+30) is degraded in a same manner as the non-inactivated PRGF membrane (mPRGF) at each study time point. Furthermore, the release kinetics of the different growth factors analyzed in the present study showed that the growth factors and proteins contained in the heat-treated mPRGF fibrin are released similarly to the mPRGF content throughout the study period. In addition, the proliferation assay performed on different ocular surface cells (HCE, HConF, and HK) using the supernatant obtained from each PRGF membrane (mPRGF and IS5+30) at each time point of the study showed no differences between them and the supernatant obtained after complete membrane squeezing (control). Furthermore, the supernatants obtained from both types of fibrin membrane exerted similar proliferative capacity on any cell type analyzed throughout the study period. These results suggest that the new heat-treated PRGF membrane (IS5+30) has similar physical and mechanical characteristics to the mPRGF and is beneficial for sustained release of proteins for long periods of time.

In addition, if we compare the results obtained in the present study with those obtained in a previous study [20] in which a heat-inactivated mPRGF was obtained directly after incubation at 56 °C for 30 min, it can be observed that no differences were found between both types of inactivated membranes (IS5+30 and is-mPRGF) in any assay carried out in both experiments. However, better mechanical properties were highlighted in the IS5+30 membrane with respect to is-mPRGF by the research personnel during the manipulation of the different membranes obtained for the performance of the different assays carried out in both studies. Although this is observational data, these results encourage us to investigate this possible difference between the two types of membrane in future studies because it could be an advantage for clinicians during the manipulation of heat-inactivated membranes in clinical practice.

## 5. Conclusions

The results obtained in the present study show the development of a new heat-treated PRGF membrane with a complete inactivation of the complement system and a reduction of IgE concentration, without reducing the levels of the main growth factors involved in tissue regeneration while preserving its biological potential. Furthermore, the present results indicate that the physical and mechanical properties of the fibrin membrane have been preserved after the heat inactivation process. Although this is a preliminary study, the results obtained in this study suggest that the new inactivated PRGF membrane could be used for the treatment of ocular surface disorders in which the immune system is related to their etiopathogenesis.

## Figures and Tables

**Table 1:** Biodegradation percentage of the different PRGF membranes after incubation with tPA for 8 days. The mPRGF and IS5+30 showed no differences in the degradation percentage at any study time.

	1 d	2 d	3 d	5 d	7 d	8 d
mPRGF + tPA	59 ± 20	45 ± 21	34 ± 16	19 ± 20	4 ± 5	0
IS5+30 + tPA	51 ± 6	26 ± 12	19 ± 11	7 ± 9	2 ± 3	0

## Data Availability

All the obtained data used to support the findings of this study are available from the corresponding author upon reasonable request.
